# Calcium in Brugada Syndrome: Questions for Future Research

**DOI:** 10.3389/fphys.2018.01088

**Published:** 2018-08-10

**Authors:** Michelle M. Monasky, Carlo Pappone, Marco Piccoli, Andrea Ghiroldi, Emanuele Micaglio, Luigi Anastasia

**Affiliations:** ^1^Arrhythmology Department, IRCCS Policlinico San Donato, Milan, Italy; ^2^Stem Cells for Tissue Engineering Lab, IRCCS Policlinico San Donato, Milan, Italy; ^3^Department of Biomedical Sciences for Health, University of Milan, Milan, Italy

**Keywords:** Brugada syndrome, sudden cardiac death, channelopathies, ion channel, fever, genetic testing

## Abstract

The Brugada syndrome (BrS) is characterized by coved-type ST-segment elevation in the right precordial leads on the electrocardiogram (ECG) and increased risk of sudden cardiac death (SCD). While it is an inheritable disease, determining the true prevalence is a challenge, since patients may report no known family history of the syndrome, present with a normal spontaneous ECG pattern at the time of examination, and test negative for all known BrS-causative genes. In fact, SCD is often the first indication that a person is affected by the syndrome. Men are more likely to be symptomatic than women. Abnormal, low-voltage, fractionated electrograms have been found in the epicardium of the right ventricular outflow tract (RVOT). Ablation of this area abolishes the abnormal electrograms and helps to prevent arrhythmic recurrences. BrS patients are more likely to experience ventricular tachycardia/fibrillation (VT/VF) during fever or during an increase in vagal tone. Isoproterenol helps to reverse the ECG BrS phenotype. In this review, we discuss roles of calcium in various conditions that are relevant to BrS, such as changes in temperature, heart rate, and vagal tone, and the effects of gender and isoproterenol on calcium handling. Studies are warranted to further investigate these mechanisms in models of BrS.

## Introduction

The Brugada syndrome (BrS) is an inherited disease characterized by coved-type ST-segment elevation in the right precordial leads on the electrocardiogram (ECG) and increased risk of sudden cardiac death (SCD) (Brugada and Brugada, 1992). BrS often presents in seemingly otherwise healthy young adults, although it has been found in patients from 2 days to 84 years old (Antzelevitch et al., 2005), and sometimes SCD (or aborted SCD) is the first indication that the patient is affected by BrS. BrS can be inherited as an autosomal dominant trait with incomplete penetrance (Chen et al., 1998; Nademanee et al., 2011; Lieve and Wilde, 2015), although in a recent study observing 135 consecutive patients with BrS, most patients did not report a family history of sudden death (Pappone et al., 2017). It is more often found in males than in females (Nademanee et al., 1997; Pappone et al., 2017) and is most prevalent in southeast Asia (Nademanee et al., 1997; Miyasaka et al., 2001; Antzelevitch et al., 2005; Napolitano and Priori, 2006; Sidik et al., 2009; Lieve and Wilde, 2015), where it is the leading cause of natural death in males under 50 years old (Brugada et al., 2005). Abnormal, low-voltage, fractionated electrograms have been found in the epicardium of the right ventricular outflow tract (RVOT). Ablation of this area abolishes the abnormal electrograms and helps to prevent arrhythmic recurrences (Pappone et al., 2017). This area, the arrhythmogenic substrate (AS), increases in size when ajmaline is administered, thus revealing the full extent of the area requiring ablation (Pappone et al., 2017). BrS patients are more likely to experience ventricular tachycardia/fibrillation (VT/VF) during fever (Martins et al., 2014) or during an increase in vagal tone (Nakazawa et al., 2003). Intravenous isoproterenol administration is beneficial for BrS patients (Kasanuki et al., 1997; Yan and Antzelevitch, 1999; Maury et al., 2004; Watanabe et al., 2006; Jongman et al., 2007). Acute myocardial ischemia of the RVOT occasionally results in a Brugada-like ECG pattern, although this is categorized as a Brugada phenocopy, rather than true BrS (Nakamura et al., 2017).

There have been excellent reviews on the pathophysiology of BrS and the proposed responsible mechanisms (Hoogendijk et al., 2010; Sieira et al., 2016). In this focused review, we discuss the role of calcium and how calcium mishandling should be investigated in BrS, especially in relation to factors that trigger arrhythmic events, such as changes in temperature, heart rate, and vagal tone. The role of gender and the beneficial effects of isoproterenol administration are also addressed. Studies are warranted to further investigate these mechanisms in models of BrS. We hope that this review stimulates debate about the role of calcium in BrS and inspires future research.

## Brugada Syndrome: Calcium and Contraction

**FIGURE 1:**
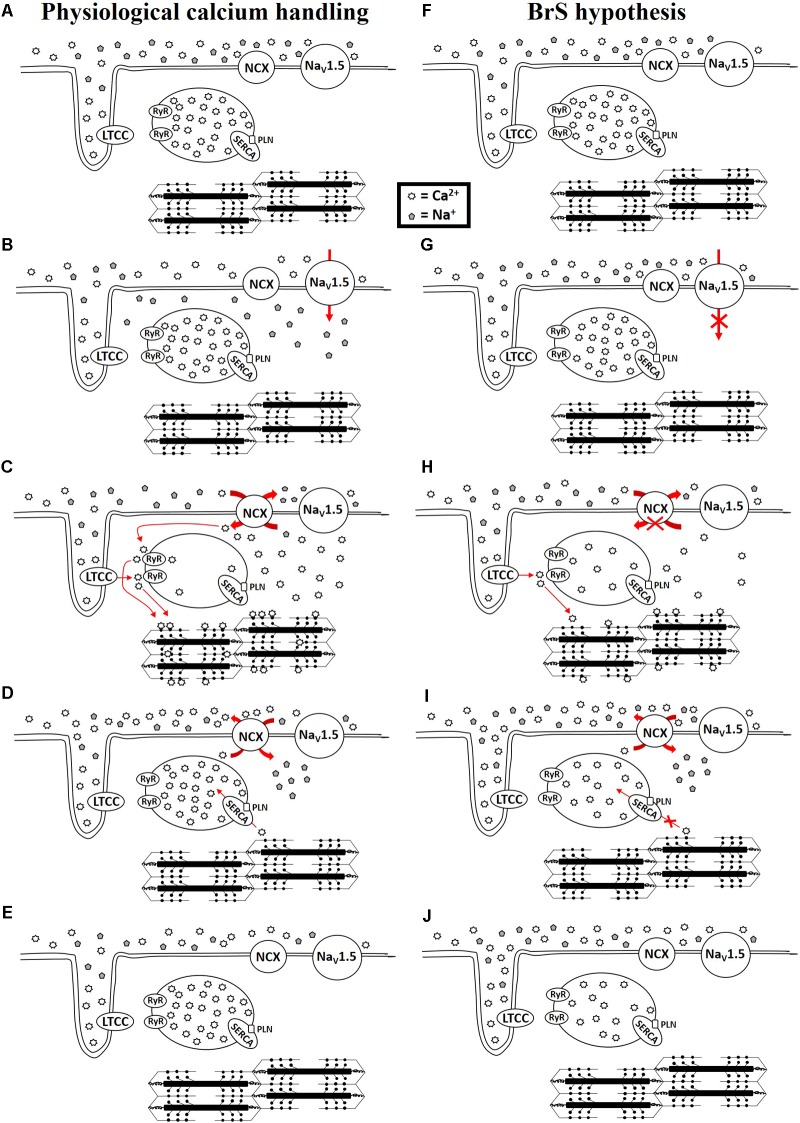
Physiological calcium handing **(A–E)** and hypothesis for calcium handing with loss-of-function of sodium channel **(F–J)** during cardiac excitation and relaxation. NCX: Na^+^/Ca^2+^ exchanger-1; Na_V_1.5: sodium channel; LTCC: L-type calcium channel; RyR: Ryanodine receptor; SERCA: sarco(endo)plasmic reticulum Ca^2+^-ATPase 2a; PLN: phospholamban.

The diagnosis of BrS is based upon the presence of a specific ECG finding, namely a coved ST-segment elevation followed by a negative T wave in the anterior right precordial leads (Brugada et al., 1993; Curcio et al., 2017). An elevated ST-segment can manifest in patients with a variety of genetic mutations, ranging from mutated ion channel proteins (Curcio et al., 2017) to mutated sarcomeric proteins (Campuzano et al., 2015; Di Resta et al., 2015; Mango et al., 2016; Monasky et al., 2017a; Song et al., 2017; Pappone, 2018), but often mutations are not detected (Curcio et al., 2017). There are several excellent reviews that discuss the various theories for the mechanism of BrS (Antzelevitch et al., 2003, 2005, 2016b; Antzelevitch, 2005; Meregalli et al., 2005; Antzelevitch and Burashnikov, 2011; Nademanee et al., 2015; Antzelevitch and Patocskai, 2016; Havakuk and Viskin, 2016). However, whether the underlying mechanism is due to conductance abnormalities arising from increased fibrosis or connexin defects or a channelopathy or sarcomeropathy, ultimately an elevated ST segment and negative T wave are produced. The ST segment and T wave correspond with phases 2 and 3 of the action potential, respectively (Paulev and Zubieta-Calleja, 1999), which correspond with intracellular calcium rise and fall, respectively (Santana et al., 2010). Antzelevitch (2002, 2006) and Antzelevitch et al. (2016a) have suggested a mechanism in BrS involving calcium depletion in cells as a consequence of reduced calcium channel current resulting from loss of the action potential. This could lead to wall motion abnormalities, particularly in the RVOT, dilation of the RVOT region, and reduced ejection fraction (EF) (Antzelevitch et al., 2016a). Moreover, sodium channel loss-of-function mutations, which occur in about 15–30% of BrS cases (Kapplinger et al., 2010), impair the function of NCX, leading to alterations in intracellular calcium (Ottolia et al., 2013). This is relevant during both systole and diastole. If there exists a loss-of-function of the sodium channels, not enough sodium will enter the cell during phase 0 to exit the cell during phase 2, which is necessary to drive the NCX to bring calcium into the cell (**Figure 1**). Calcium that normally enters the cell during phase 2 via NCX triggers additional sarco(endo)plasmic reticulum (SR) calcium release via activation of the ryanodine receptors. Therefore, reduced calcium influx via NCX may lead to reduced SR calcium release. During diastole, reductions in intracellular calcium may result in a loss of calcium from the cell as the SR is unable to effectively compete with NCX for calcium uptake. A reduction in SR calcium store would ultimately lead to a reduction in contractility. Therefore, a loss-of-function of the sodium channel, which occurs in many BrS patients, would impair physiological calcium handling, although to what extent is uncertain. Abnormalities in calcium signaling (increase/decrease in calcium transient amplitude or alterations in kinetics of calcium transport) result in changes in the amount of calcium available to bind to troponin C of the myofilaments to induce or sustain contraction. Therefore, disruptions to the action potential can lead to calcium mishandling and disrupted excitation-contraction coupling. Thus, BrS patients are at higher risk of experiencing contractile dysfunction than people without BrS.

Reduced contractility in BrS is supported by several studies. Catalano et al. (2009) described RVOT diameter, area, and volume to be similar in BrS and control patients, but RVOT EF was decreased in BrS patients due to an increased end systolic volume (ESV). Tukkie et al. (2004) demonstrated that RV ejection time shortens as the ECG BrS pattern develops during flecainide challenge, suggesting reduced calcium influx. Rudic et al. (2016b) reported significantly lower RVEF, as well as a trend toward lower LVEF, in BrS patients compared with healthy controls. Papavassiliu et al. (2010) reported patients with a spontaneous type 1 BrS ECG with significantly lower RVEF and ESV compared to controls. van Hoorn et al. (2012) described patients with the SCN5A sodium channel mutation having significantly larger end diastolic volume (EDV) and ESV in the RV and LV compared to patients without the SCN5A mutation or volunteers. Thus, contractility is reduced in BrS, which, accompanied by altered ST segment and T wave ECG recordings, suggests altered calcium handling during excitation-contraction coupling. Studies are warranted to further investigate calcium signaling in BrS.

In addition to contractile dysfunction, BrS is associated with structural abnormalities. Miyazaki et al. (1996) named the presence of right bundle branch block, ST segment elevation, and ventricular tachyarrhythmias “Brugada syndrome.” This was after Brugada and Brugada (1992) had described right bundle branch block, normal QT interval, and persistent ST segment elevation in precordial leads V1 to V2–V3 in the absence of echocardiographic or histologic abnormalities as being a distinct syndrome different from idiopathic ventricular fibrillation. However, before that, in Martini et al. (1989) had described six patients resuscitated from ventricular fibrillation. An upsloping ST-T segment elevation (“early repolarization”) was observed in three patients in V1, V2. Stimulation of the RVOT during electrophysiological study triggered ventricular fibrillation. Although structural heart disease had been initially excluded, abnormalities, predominantly of the right ventricle, were realized after a more in-depth assessment. Fibrosis was detected in three of the patients. Half of the patients were at rest when the ventricular fibrillation occurred, four out of six had no preexisting symptoms, and five out of six had no documented arrhythmias. The group concluded that electrical heart disease may often be associated with a pathological substrate predominantly in the right ventricle, and that greater attention should be given to ECG patterns of right bundle branch block, early repolarization (ST segment elevation), inverted T waves, and late QRS potentials (Martini et al., 1989) [also see letter arguing for the existence of structural abnormalities (Martini et al., 1993)]. Nevertheless, a great amount of literature over the following decades referred to BrS as an electrical disease without structural abnormalities. More recently, the dogma surrounding this issue has reversed to recognize that in fact structural abnormalities exist. BrS is now accepted to be associated with epicardial surface and interstitial fibrosis (Coronel et al., 2005; Nademanee et al., 2015), reduced gap junction expression, and increased collagen (Nademanee et al., 2015). Right ventricular histological samples from BrS patients have exhibited myocarditis, cardiomyopathic changes, or myocardial fatty infiltration typical of arrhythmogenic right ventricular cardiomyopathy (ARVC) (Frustaci et al., 2005). These studies support earlier ones, such as the one by Corrado et al. (2001) which aimed to assess the prevalence of right precordial ST-segment elevation in young sudden death victims, and found that this ECG pattern was strongly correlated with RV structural abnormalities consistent with ARVC involving predominantly the RV anterior wall. While little is known about calcium handling in BrS, structural cardiomyopathies such as ARVC are associated with significant pathological calcium handing (for an excellent review, see van Opbergen et al., 2017). In short, the field now recognizes that BrS is associated with structural abnormalities. Therefore, what is already known about calcium mishandling in these pathologies (i.e., ARVC, myocarditis, etc.) could be applied as a starting point to hypothesize about calcium mishandling in BrS.

## The Genetics of Brugada Syndrome: Ion Channel, Sarcomeric, and Mitochondrial Mutations

Brugada syndrome cases are consistently familial: BrS exhibits an autosomal dominant pattern of inheritance with equal genetic transmission to both genders. Recent technical improvements have changed BrS molecular diagnostic testing capabilities. Next generation sequencing allows a cost-effective genetic analysis. Using this method, nearly 300 pathogenic variants in 22 different genes have been published to date. The genes today associated with BrS can codify for sodium channels, sodium channel regulators, sarcolemma associated proteins, potassium channels, calcium channels, trafficking regulators, and signaling proteins (Kapplinger et al., 2010; Di Resta et al., 2015).

The three most common types of genetic variations found in BrS patients are missense, nonsense, and splicing mutations. Excellent reviews have been written on this subject (Kapplinger et al., 2010; Wilde and Amin, 2018). A missense mutation is a change in one DNA base pair that results in the substitution of one amino acid for another in the protein codified by a certain gene (Skinner and Love, 2010). In medical literature, it is well known that missense mutations are found in almost two thirds of BrS patients who are found to have a mutation during genetic screening (Kapplinger et al., 2010). Missense mutations in SCN5A are an important molecular cause of BrS (Kapplinger et al., 2010). For example, missense heterozygous mutations like c.1066G > A (p.Asp356Asn) or c.4222G > A (p.Gly1408Arg) are observed in BrS patients. Loss-of-function missense mutations have been described in CACNA1C (A39V and G490R) and CACNB2 (S481L), which encode the alpha1- and beta2b-subunits of the L-type calcium channel (Antzelevitch et al., 2007). These calcium channel mutations have been identified in BrS patients who also exhibit shorter-than-normal QT intervals (Antzelevitch et al., 2007).

In a nonsense mutation, the altered DNA sequence prematurely signals the cell to stop producing a protein. This type of mutation results in a shortened protein that may function improperly or not at all. A subgroup of BrS patients harbor a heterozygous nonsense mutation, most commonly in the SCN5A gene (Teng et al., 2017). Two examples of this kind of mutation are c.4867C > T (p.Arg1623Ter) and c.5435C > A (p.Ser1812Ter), both associated with serious functional alterations in the Na_V_1.5 protein, codified by the SCN5A gene.

Splicing is the editing of precursor messenger RNA into mature messenger RNA, removing non-coding regions (called “introns”) and joining together all coding regions (Qin et al., 2018). Theoretically, every kind of mutation can affect the splicing process, for example with exon skipping, intron retention, or cryptic splice site activation (Peccarelli and Kebaara, 2014). Fukuyama et al. (2014) reported an important example of this in 2014. They described a missense heterozygous mutation in the CACNA1C gene (c.1896G > A, p. Arg632Arg) causing an exon skipping in mRNA.

A less common type of mutation found in BrS patients is the frameshift mutation, which occurs when the addition or loss of DNA bases changes a gene’s reading frame. A reading frame consists of groups of three bases, each coding for one amino acid. A frameshift mutation shifts the grouping of these bases and changes the code for amino acids. The resulting protein is usually nonfunctional. A clear example of this in BrS can be found in the heterozygous mutation D1816VfsX7 in the SCN5A gene (Dolz-Gaiton et al., 2013). Insertions, deletions, and duplications can all be frameshift mutations and have been described in BrS (Mobius-Winkler et al., 2011). An insertion changes the number of DNA bases in a gene by adding a piece of DNA. As a result, the protein made by the gene may not function properly. A deletion changes the number of DNA bases by removing a piece of DNA. Small deletions may remove one or a few base pairs within a gene, while larger deletions can remove an entire gene or several neighboring genes. The deleted DNA may alter the function of the resulting protein(s). A duplication consists of a piece of DNA that is abnormally copied one or more times. In medical literature, large deletions involving both the SCN5A and SCN10A genes are described as an emerging cause of BrS (Sonoda et al., 2018).

In one report, a Brugada phenotype was described as X-linked, likely caused by a mutation in the KCNE5 gene (Ohno et al., 2011). It is sufficient to harbor a single copy mutation in a diseased gene to be affected by BrS, with a 50% recurrence risk if molecularly confirmed. The most commonly mutated gene in BrS is SCN5A (Di Resta et al., 2015; Sieira et al., 2016; Curcio et al., 2017), which encodes for the alpha subunit of the cardiac sodium channel (Di Resta et al., 2015), although a mutation in one of several genes can be causative of BrS (**Table 1**). Pathogenic variants in SCN5A are associated with a functional impairment, not only in the sodium channel (Calloe et al., 2013), but also in the whole heart (van Hoorn et al., 2012). Mutations in the SCN5A gene have been found responsible for ARVC, atrial standstill type 1, atrial fibrillation, left ventricular noncompaction, dilated cardiomyopathy, Long QT syndrome type 3, sick sinus syndrome type 2, idiopathic ventricular fibrillation, and heart block type 1A (Gosselin-Badaroudine et al., 2014; Zaklyazminskaya and Dzemeshkevich, 2016). Calcium channel mutations in CACNA1C, CACNB2b, and CACNA2D1 are all associated with loss-of-function of the channel and have been extensively reviewed previously, including the loss-of-function mutations that occur in these genes in Short QT Syndrome and Early Repolarization Syndrome (Betzenhauser et al., 2015).

**Table 1:** Mutated genes used to molecularly confirm BrS.

Genes	Patients	Reference
*CACNA1C, CACNB2*	82	Antzelevitch et al., 2007
*CACNA1C, CACNA2D1, CACNB2*	162	Burashnikov et al., 2010
*SCN5A*	2111	Kapplinger et al., 2010
*KCNJ8*	204	Barajas-Martinez et al., 2012
*ABCC9*	150	Hu et al., 2014
*AKAP9, ANK2, KCNJ2, CASQ1, RYR2*	45	Allegue et al., 2015
*KCNH2, ANK2, SCN5A, RYR2*	16	Farrugia et al., 2015
*SCN5A*	1	Chen et al., 2016
*CACNA1C*	1	Bai et al., 2017
*SCN1B*	23	Gray et al., 2018

Understanding the effects of each mutation is quite complex, especially when an individual BrS patient harbors pathogenic variants in two or more genes. In addition, pathogenic variants in SCN1B, SCN2B, and SCN3B genes can act as disease modifiers rather than as a single molecular cause of BrS (Watanabe et al., 2008; Riuro et al., 2013). Furthermore, BrS can coexist with other phenotypes within the same individual, such as Long QT syndrome type 3 or Lev-Lenègre disease (Meregalli et al., 2005). It is very common that family members with the same mutation can have different phenotypes between members of that family [left ventricular noncompaction or BrS (Pappone, 2018); BrS in males but Lev-Lenègre disease in females (Kyndt et al., 2001; Meregalli et al., 2005)]. Future studies are warranted to better understand how specific mutations result in different phenotypes between individuals. In particular, it is already unclear how females with BrS can be asymptomatic more frequently than males and how important genetic modifiers can explain the incomplete penetrance and clinical variability of BrS familial cases. One study examining the role of mitochondrial DNA used PCR-SSCP analysis to describe four recurrent mutations in mitochondrial tRNA genes associated with BrS for the first time (Tafti et al., 2018). This suggested that mitochondrial DNA may lead to functional deficiencies in the translational process of myocardial proteins (i.e., a frameshift mutation). This can lead to nonfunctioning proteins important in BrS.

Although BrS has commonly been described as a channelopathy, several studies have now identified sarcomeric mutations with the syndrome (Ishikawa et al., 2012; Campuzano et al., 2015; Di Resta et al., 2015; Mango et al., 2016; Monasky et al., 2017a; Song et al., 2017; Pappone, 2018), suggesting BrS may originate as a sarcomeropathy. Like many forms of arrhythmia and cardiomyopathy, BrS can originate from a variety of mechanisms. Thus, there may be no one underlying mechanism for BrS, but likely there are several mechanisms that ultimately converge into one common pathway that results in a particular recognizable phenotype. Thus, the question when sarcomeric mutations are detected becomes: could sarcomeric mutations result in a BrS phenotype?

While sarcolemmal calcium transport proteins clearly play a vital role in calcium transport and thus activation of the myofilaments, it has long been established that contractile force and relaxation are not merely regulated by passive binding and unbinding of calcium to the myofilaments, but that the myofilaments independently regulate this process (Monasky et al., 2008a, 2017b). This can occur, for example, through genetic mutations (Baudenbacher et al., 2008; Yar et al., 2013; Alves et al., 2014; van der Velden et al., 2015) or post-translational modifications (Varian et al., 2009; Monasky et al., 2010, 2013; Lovelock et al., 2012) of the myofilaments that alter myofilament calcium sensitivity (MCS). In pulmonary artery banded rabbits, mechanical strain placed on the myofilaments feeds back to regulate calcium signaling by decreasing expression of the SR Ca^2+^-ATPase (SERCA) 2a and increasing expression of the Na^+^/Ca^2+^ exchanger-1 (NCX) (Gupta et al., 2009). Mutated troponin T leading to increased MCS in mice results in arrhythmia susceptibility (Baudenbacher et al., 2008; Yar et al., 2014). It has been suggested that calcium sensitization causes a substrate for reentrant activation (Huke and Knollmann, 2010). The role of increased MCS on arrhythmia susceptibility has been extensively reviewed elsewhere (Huke and Knollmann, 2010). Future studies are warranted to understand MCS and its role in arrhythmia susceptibility in BrS. This is particularly interesting in individuals with overlap diseases, such as hypertrophic cardiomyopathy, dilated cardiomyopathy, or ARVC in addition to BrS (Gray et al., 2014; Steiner et al., 2014; Mango et al., 2016; Monasky et al., 2017a), or families in which different family members with the same sarcomeric mutation exhibit diverse phenotypes, including BrS and another cardiomyopathy (Pappone, 2018).

### Genetic Models

Considering up to 30% of BrS cases have been mainly associated with a genetic mutation in the SCN5A gene, (London et al., 2007) the creation of animal models carrying these mutations is very useful for the characterization of the pathophysiology of BrS. Park et al. (2015) have described a pig model with a SCN5A nonsense mutation (E558X) previously found in a child with BrS. This model exhibited slowed conduction and increased susceptibility to ventricular arrhythmias, and presents a promising model for future BrS studies. Indeed, the creation of a mutant pig model, which has a more similar cardiac structure and function to humans than smaller animal models, is extremely useful to understanding BrS. The action potential and calcium handling are known to greatly differ between species, thus making a larger animal model preferable to understanding BrS in humans (Milani-Nejad and Janssen, 2014).

Murine models have also been used. Although the action potentials and calcium handling differ greatly between mice and humans, these models can be more quickly and cost-effectively created, are easier to handle and house, and have provided a great deal of knowledge. Thus, murine models are preferred by a number of groups. Indeed, a murine heterozygous SCN5A+/− model was generated for BrS *in vivo* studies (Papadatos et al., 2002). This model has been used by several groups to mimic BrS, demonstrating how the SCN5A haploinsufficiency was responsible for the onset of a complex range of phenotypes, including a 50% reduction of sodium conductance, a delayed intramyocardial conduction, a sinus node dysfunction, and ventricular arrhythmogenesis, which are hallmarks of BrS patients (Zhang et al., 2014; Tse et al., 2017; Kelly et al., 2018). However, caution must be made when translating the results of action potential studies in murine models to the situation in humans, as many differences exist between the species (Milani-Nejad and Janssen, 2014).

Some kinds of genomic rearrangements, such as deletion or duplication, in the SCN5A gene can cause BrS as well (Sonoda et al., 2018). Alternative models must be made for the remaining ∼70% of patients who do not carry a SCN5A mutation. Moreover, BrS has been currently associated with “new” genetic mutations (Antzelevitch and Patocskai, 2016). Thus, creating informative animal models is a challenge because: (a) it is unclear which is the main mutation involved in the onset of the pathology, and (b) these models should develop the same phenotype, independently from the different genetic alterations. Therefore, it would be ideal to start with more comprehensive human genomic studies. Comparative studies between affected individuals from different races, such as East Asian vs. Caucasian, could help elucidate which mutations are actually responsible for the phenotype, since there is a higher prevalence observed clinically in East Asian countries. Additionally, the impact of sarcomeropathies in BrS remains an open question.

## The Arrhythmogenic Substrate

Several studies have described the AS responsible for abnormalities seen in the ECG in BrS to be located in the RVOT (Lambiase et al., 2009; Nademanee et al., 2011; Sunsaneewitayakul et al., 2012; Cortez-Dias et al., 2014; Rudic et al., 2016a). Studies in rabbit myocytes suggest this may be partially explained by a wider range of action potential durations (APDs) in RVOT myocytes compared to RV myocytes, which is an important factor in arrhythmogenesis (Liang et al., 2012). Prolongation of the APD in RVOT myocytes can be caused in part by higher current density of I_Ca-L_, which normally occurs in RVOT myocytes (Liang et al., 2012). This suggests that RVOT myocytes normally rely more heavily on L-type calcium channel current, which would suggest that the RVOT relies more heavily on the SR calcium that the increased L-type calcium channel current would trigger, compared to RV myocytes. Differences in the embryological origins of RVOT and RV cardiomyocytes may help explain these differences (Liang et al., 2012), as the RVOT may have retained embryonic features as it originated from the more slowly conducting embryonic outflow tract (Rana et al., 2007; Boukens et al., 2013). The transversal conduction velocity is further reduced in the RVOT compared to the RV in healthy myocardium after ajmaline administration (Boukens et al., 2013). In a mouse model of BrS, with a 1798InsD mutation in SCN5A, conduction velocity was reduced in both RVOT and RV compared to wild type, but was more pronounced in the RVOT, suggesting the continued presence of the embryonic gene program (Boukens et al., 2013). Demembranated human embryonic stem cell-derived cardiomyocytes (d-hESC-CMs) exhibit reduced maximal generated force, increased calcium sensitivity, faster kinetics of force re-development at submaximal activation levels, and faster kinetics of the slow relaxation phase compared to adult human ventricular myofibrils, probably due to differential sarcomeric protein isoform expression (Iorga et al., 2017). Future studies could also investigate the distribution of calcium cycling related genes at the mRNA and/or protein levels compared to other anatomical positions in the heart. Further studies are warranted to understand why and how the AS originates at the RVOT in BrS.

## Ajmaline to Diagnosis and Research Brugada Syndrome

A sodium channel blocking agent, such as ajmaline (Yap et al., 2009; Dobbels et al., 2016), is used to provoke the type 1 BrS ECG pattern to affirmatively diagnose the syndrome in patients with spontaneous type 2, type 3, or normal ECG patterns (Antzelevitch et al., 2016b; Havakuk and Viskin, 2016; Rudic et al., 2016a). Together with epicardial mapping, ajmaline challenge can reveal the extent of the AS (Pappone et al., 2017). Prolonged fragmented ventricular potentials have been used to define the extent of the AS in BrS (Pappone et al., 2017). VT/VF is a possible side effect of ajmaline administration (Conte et al., 2013; Dobbels et al., 2016). Care must be made to watch a patient’s response to the ajmaline administration, including using a slow infusion rate and stopping the administration early in patients whose ECG changes rapidly (Arnalsteen-Dassonvalle et al., 2010). Thus, ajmaline aids to uncover and visualize the extent of the AS. Understanding better the mechanism of ajmaline could lead to better understanding of the mechanism of BrS. For example, what are the effects of ajmaline on potassium and calcium signaling? What post-translational modifications of ion signaling proteins and contractile proteins occur as a result of ajmaline administration? How does ajmaline provoke a type 1 BrS pattern even in patients negative for sodium channel mutations? It would be particularly interesting to see the effects of ajmaline on proteins involved in the β-adrenergic signaling pathway, since isoproterenol reverses the effects of ajmaline. Thus, further studies are warranted to better understand the mechanism of ajmaline.

## Fever and Brugada Syndrome: Calcium, Temperature, and Heart Rate

**FIGURE 2:**
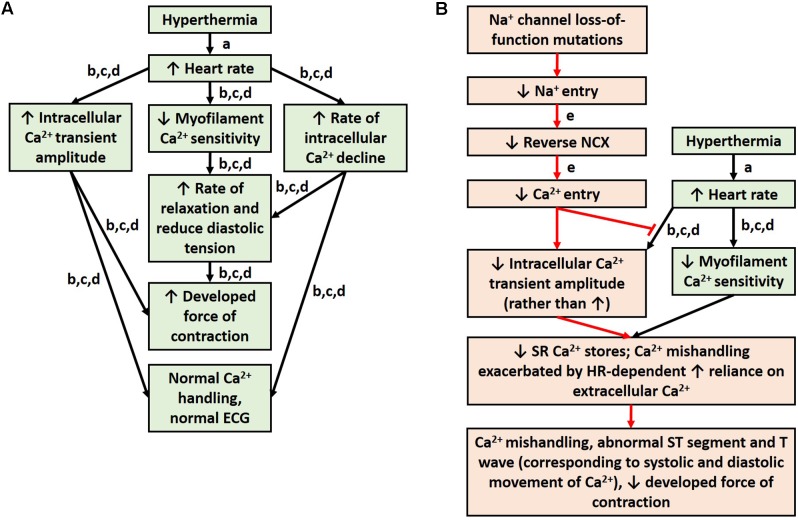
Physiological response to hyperthermia **(A)**. Hypothesis for mechanism of ECG abnormalities during fever in patients with sodium loss-of-function mutations **(B)**. References: a (Karjalainen and Viitasalo, 1986); b (Hiranandani et al., 2006); c (Varian and Janssen, 2007); d (Varian et al., 2009); and e (Ottolia et al., 2013).

Brugada syndrome patients are more likely to experience T wave abnormalities and VT/VF during a febrile state (Martins et al., 2014). Normally, hyperthermia is accompanied by an increase in heart rate (Karjalainen and Viitasalo, 1986). Studies from healthy rabbit and rat myocardium indicate that an increase in heart rate alone causes an increase in intracellular calcium amplitude, an increase in developed force of contraction, and decreased MCS (Hiranandani et al., 2006; Varian and Janssen, 2007) (**Figure 2A**). The decrease in MCS allows the myocardium to relax faster at high heart rates to ensure adequate refilling of the heart chamber before the next contraction and is countered by an increase in intracellular calcium, which results in a net inotropic effect (Varian and Janssen, 2007; Varian et al., 2009). Experiments in rats demonstrate that an increase in temperature, however, diminishes this inotropic effect of heart rate (Hiranandani et al., 2006). In fact, higher temperatures reduce the force of contraction despite a rise in intracellular calcium (Hiranandani et al., 2006). This suggests a reduction in MCS from an increase in temperature alone, which could be exacerbated at high heart rates, which also independently reduce MCS. Antzelevitch and colleagues (Antzelevitch, 2002, 2006; Antzelevitch et al., 2016a) have suggested a mechanism in BrS involving calcium depletion in cells as a consequence of reduced calcium channel current resulting from loss of the action potential. If in fact calcium depletion occurs in BrS, contractile malfunction may become more evident at higher heart rates when calcium handling proteins are stressed to cycle calcium more quickly, and the reductions in cytosolic calcium become more significant (Varian et al., 2009). Cells would have difficulty drawing the extra calcium from the SR to produce the increase in intracellular calcium amplitude that is normally seen at higher heart rates. Additionally, channelopathies, such as loss-of-function of sodium channels, can prevent physiological alterations in calcium handling that are normally seen with changes in temperature or heart rate, since calcium signaling is dependent on sodium signaling (**Figure 2B**). Furthermore, reduced cytosolic calcium could reduce contractility more noticeably at higher heart rates due to the reduced MCS. It would be interesting for future studies to investigate calcium signaling and store in BrS and how this is altered with changes in temperature and heart rate. It would be particularly interesting to investigate the mechanisms involved in myocardial relaxation and intracellular calcium decline, since alterations in T wave tracings are seen in feverish BrS patients, and T waves correspond to phase 3 of the action potential (Paulev and Zubieta-Calleja, 1999), when intracellular calcium is being transported out of the cytosol. Due to the alterations in MCS that occur during higher temperatures and heart rate and the ability of myofilament calcium buffering to affect calcium uptake, alterations to the sarcomeric proteins would also be of interest for future studies.

## Vagal Tone and Risk of Arrhythmias in Brugada Syndrome

The cardiac parasympathetic (vagal) nerves, the sympathetic nerves, and the pacemaker cells are essentially the factors that contribute to heart rhythm (Coote and White, 2015). In a review by Hou et al. (2016) the authors have noted that increased vagal tone or decreased sympathetic function may worsen arrhythmogenesis in patients with J-wave syndrome, including BrS, contrary to most other ventricular tachyarrhythmias. In fact, several BrS studies suggest an association between an increase in vagal tone and an increased risk for arrhythmic events (Nakazawa et al., 2003), which may occur while resting, sleeping (Mizumaki et al., 2006; Takigawa et al., 2008; Lieve and Wilde, 2015), after a large meal (Lieve and Wilde, 2015), or while vomiting (Arai et al., 2004). Parasympathetic stimulation results in an elevated ST segment, and it has been suggested the mechanism is through a reduction in I_Ca−L_ during the action potential plateau (Litovsky and Antzelevitch, 1990; Meregalli et al., 2005). Enhanced vagal tone can also lead to reduced heart rate (Coote and White, 2015; Ponganis et al., 2017). Studies from healthy rat and rabbit myocardium indicate that a decrease in heart rate, in turn, results in a decrease in intracellular calcium amplitude, a decrease in developed force of contraction, and increased MCS (Hiranandani et al., 2006; Varian and Janssen, 2007). Thus, lower heart rates are associated with less intracellular calcium, and a reduction of calcium current is associated with the development of an elevated ST segment. It would be interesting to study this mechanism further in models of BrS to understand the level of calcium present in the cell before parasympathetic stimulation, as well as the role of calcium during parasympathetic stimulation.

## Perceived Prevalence, Genetic Transmission, and Hormone Regulation

Brugada syndrome is more often found in males than in females (Nademanee et al., 1997; Pappone et al., 2017), despite equal genetic transmission to both genders (Meregalli et al., 2005). BrS signs and symptoms can manifest in adulthood in patients who previously tested ajmaline negative and who had normal ECGs in childhood, including patients with or without a genetic mutation associated with BrS (Conte et al., 2014), suggesting a role for hormones in BrS. In another report, the typical BrS ECG pattern disappeared in two patients who underwent surgical castration for prostate cancer, suggesting a role for testosterone (Matsuo et al., 2003). Gender impacts autonomic control of the cardiovascular system, including vagal modulation and heart rate, and thus may play an additional role in the mechanisms discussed earlier in this article (Mann et al., 2012; Pothineni et al., 2016). Estrogen upregulates L-type calcium channels, and thus plays a role in calcium handling (Yang et al., 2012), which is important in BrS as described earlier above. Although contractile performance and β-adrenergic response has been reported to be not significantly different between the genders in healthy isolated rat cardiac trabeculae (Monasky et al., 2008b), there are gender differences in ion channel and transporter subunit expression in non-diseased human hearts (Gaborit et al., 2010), suggesting there could be a role for gender in disease states in which calcium handling is pathologically altered. Hormone regulation of cell communication and differences in ion transport are thus important topics for future research in BrS. Future studies should investigate how hormone regulation may place men at greater risk generally, making it easier for them to be diagnosed, and how gender alters the risk of arrhythmic events during triggering factors (i.e., fever, decreased vagal tone), making it even easier for men to be diagnosed.

## β-Adrenergic Response, Isoproterenol Use in Brugada Syndrome Patients

Several studies have reported that intravenous isoproterenol administration reduces ST elevation in leads V1 to V3 and restores the action potential dome in BrS experimental models and patients by increasing calcium current (Kasanuki et al., 1997; Yan and Antzelevitch, 1999; Maury et al., 2004; Watanabe et al., 2006; Jongman et al., 2007). Jongman et al. (2007) have further reported a patient who experienced a “clear positive result on the recurrence of VF” after isoproterenol administration, while no increase in heart rate was observed, concluding that the therapeutic mechanism was likely a direct effect on the myocytes, rather than a beneficial effect from a higher heart rate. β-adrenergic stimulation by isoproterenol increases SR calcium content and increases the rate of calcium transient decay by phosphorylating phospholamban, relieving its inhibition on SERCA (Wegener et al., 1989; Neumann et al., 1995; Eisner et al., 2000; Grimm and Brown, 2010). β-adrenergic receptor stimulation also leads to the phosphorylation of cardiac myosin binding protein C and troponin I (Neumann et al., 1995; Grimm and Brown, 2010), which leads to reduced MCS, faster release of calcium from the myofilaments, and faster relaxation. Thus, in addition to increasing calcium current, isoproterenol administration leads to an increased SR calcium content and a shorter diastole through PKA activation, resetting the cell for the next activation. Furthermore, PKA activated by β-adrenergic receptor stimulation leads to an acute time-dependent increase of sodium current (Abriel, 2007), which would help the cell to accumulate calcium (Ottolia et al., 2013). Future experiments are warranted to investigate the calcium handing and SR calcium store in BrS, as well as the effect of isoproterenol on calcium handling in these models in the presence of arrhythmogenic triggering factors. An interesting hypothesis would be that isoproterenol works by restoring calcium to physiological levels in BrS. This would be consistent with the finding that β-blockers, which work to block the β-adrenergic response, are not effective for BrS patients, as reported by Brugada et al. (1998).

## Brugada Phenocopy During Ischemia

Brugada phenocopies (BrPs) describe conditions that produce Brugada-like ECG patterns but are generated by other clinical or technical factors (Baranchuk et al., 2012; Anselm et al., 2014; Agrawal et al., 2015). One such BrP occasionally occurs during acute myocardial ischemia of the RVOT (Nakamura et al., 2017). Nakamura et al. demonstrated that this type of BrP predominantly occurs in male patients and that subsequent development of VT/VF may result (Nakamura et al., 2017). Interestingly, in the same study, VT/VF was reported in patients without any obstructive lesion, but arrhythmic events were not detected when preceded by coronary occlusion or stenosis of the conus or RV branch, suggesting an effect of preconditioning (Nakamura et al., 2017). In rats, preconditioning with isoflurane prevents ischemia/reperfusion-related degradation of RyR and SERCA (An et al., 2007). Eisner et al. have described a role for SR calcium in ischemia (Eisner et al., 2000). During ischemia, decreases in [ATP] hinder SERCA activity and thus calcium uptake into the SR (Eisner et al., 2000). Despite this, an inhibitory effect on also the RyR may result in the net increase in the SR calcium store (Eisner et al., 2000). This decrease in both release and uptake of calcium from the SR suggests a reduced role for SR calcium in the activation and relaxation of the myocardium. Thus, alterations in the cell’s reliance on SR calcium during ischemia may mimic on clinical exams other situations in which the cell’s reliance on SR calcium is altered. Since calcium plays a significant role in ischemia, which can result in a BrP, it would be interesting to further study the role of RyR and SERCA activity in BrS. Furthermore, as mentioned above, it has been suggested that a reduced I_Ca-L_ during the action potential plateau can contribute to an elevated ST segment during parasympathetic stimulation (Meregalli et al., 2005). Thus, the role of calcium and how it can contribute to an elevated ST segment in BrS should be further investigated. Additionally, the finding that BrP during acute myocardial ischemia predominantly occurs in males lends further support for future studies on the role of hormones in ion transport regulation in BrS.

## Future Directions

Future studies should aim to better understand the genetics of BrS so as to enable researchers to create models that represent the approximately 70% of patients who do not harbor a SCN5A mutation. It is important also to investigate how hormone regulation could lead to a seemingly greater prevalence in men, how hormone regulation affects ion signaling, and how gender influences cellular processes during changes in temperature, heart rate, and vagal tone. Studies are warranted to understand why and how the AS originates at the RVOT in BrS, including expression levels of ion transport proteins and the post-translational modification state of those proteins. It would also be interesting to further understand the mechanism of ajmaline, for example, if ajmaline administration alters the post-translational modification status of key ion transport, contractile, or gap junction proteins, particularly the same proteins affected by the β-adrenergic response.

Donor hearts or animal models could be used to investigate intracellular sodium, potassium, and calcium signaling, as well as MCS and SR calcium store. Also, changes in post-translational modification status and expression level of key regulatory proteins could be studied, such as phosphorylation levels of proteins in the β-adrenergic signaling pathway and SERCA and phospholamban expression levels.

Another attractive tool for studying the pathogenic mechanisms of BrS could be the use of induced-Cardiomyocytes (iCMs), which have been recently derived from induced-Pluripotent Stem Cells (iPSCs), or through the direct reprogramming of fibroblasts. Indeed, a few groups have already generated and used iCMs to perform electrophysiological studies at the cellular level to characterize BrS ion current alterations. In particular, iCMs obtained from fibroblasts from BrS patients carrying an SCN5A mutation exhibited BrS features, including a reduction in inward sodium current, a shift in voltage-dependence curves and abnormal calcium handling (Liang et al., 2016; Selga et al., 2018). Remarkably, iCMs generated from BrS patients without SCN5A mutations showed no significant functional difference when compared to iCMs generated from healthy controls (Miller et al., 2017; Koivumaki et al., 2018). These results highlight the limitations of these studies, as clearly the BrS phenotype is observed clinically in patients who test negative for SCN5A mutations. Indeed, while iCMs possess most of the features of adult cardiomyocytes, they still show an important functional and electrical immaturity, accompanied also by morphological and structural characteristics typical of embryonic cardiomyocytes. This aspect still represents a major issue for iCMs use as an *in vitro* model of adult cardiomyocytes, especially when considering that ion channels are greatly modified during embryogenesis and development (RIF).

## Conclusion

Many questions remain to understand the clinical presentation of BrS. Further human genomic studies are warranted to better understand the commonalities between BrS patients. Additional animal and cellular models are required to clarify the questions outlined in this review, and much effort is needed to improve their consistency and reproducibility before they can be considered as effective tools to study BrS.

## Author Contributions

MM conceived the review, wrote the initial draft, edited the final draft, and approved the final version. CP, MP, AG, EM, and LA helped for the concept discussion, edited the final draft, and approved the final version.

## Conflict of Interest Statement

The authors declare that the research was conducted in the absence of any commercial or financial relationships that could be construed as a potential conflict of interest. The reviewer BJB and handling editor declared their shared affiliation at the time of the review.
